# Electrical Signatures of Diffusion-Limited Mixing: Insights from a Milli-fluidic Tracer Experiment

**DOI:** 10.1007/s11242-021-01607-0

**Published:** 2021-05-24

**Authors:** Alejandro Fernandez Visentini, Pietro de Anna, Damien Jougnot, Tanguy Le Borgne, Yves Méheust, Niklas Linde

**Affiliations:** 1 Institute of Earth Sciences, University of Lausanne, CH-1015 Lausanne, Switzerland; 2 Université de Rennes 1, CNRS, Géosciences Rennes UMR 6118, 35042 Rennes, France; 3 Sorbonne Université, CNRS, EPHE, UMR 7619 METIS, Paris, France

**Keywords:** Upscaled electrical conductivity, Diffusion-limited mixing, Hydrogeophysics, Milli-fluidic experiment

## Abstract

We investigate how diffusion-limited mixing of a layered solute concentration distribution within a porous medium impacts bulk electrical conductivity. To do so, we perform a milli-fluidic tracer test by injecting a fluorescent and electrically conductive tracer in a quasi two-dimensional (2D) water-saturated porous medium. High resolution optical- and geoelectrical monitoring of the tracer is achieved by using a fluorimetry technique and equipping the flow cell with a resistivity meter, respectively. We find that optical and geoelectrical outputs can be related by a temporal re-scaling that accounts for the different diffusion rates of the optical and electrical tracers. Mixing-driven perturbations of the electrical equipotential field lines cause apparent electrical conductivity time-series, measured perpendicularly to the layering, to peak at times that are in agreement with the diffusion transport time-scale associated with the layer width. Numerical simulations highlight high sensitivity of such electrical data to the layers’ degree of mixing and their distance to the injection electrodes. Furthermore, the electrical data correlate well with time-series of two commonly used solute mixing descriptors: the concentration variance and the scalar dissipation rate.

## Introduction

Time-lapse direct-current (DC) geophysical data acquired during saline tracer tests in porous media can provide constraints, both in the field and in the laboratory, on the temporal evolution of solute mixing processes. DC electrical resistance data are acquired using electrode configurations consisting of two electrode pairs: one to drive a known electrical current between two positions (points, along lines or across surfaces depending on application) and another to measure the voltage between two other positions (e.g., Keller and Frischknecht 1966). When considering the injected current and the measurement geometry, these voltages are readily transformed into apparent resistivities. The number of electrode configurations used is sometimes few, for example, when considering the equivalent electrical conductivity (inverse of resistivity) tensor of small-scale laboratory core samples (e.g., Maineult et al. 2016; Ghosh et al. 2018), and sometimes large (hundreds or many thousands) for field experiments aiming at inferring the spatial distribution of bulk electrical resistivity; a process often referred to as electrical resistivity tomography (ERT). When applied in time-lapse mode, DC measurements are repeated over time to produce time-series of apparent resistivities. See the recent book by Binley and Slater (2021) for a comprehensive overview.

The bulk electrical conductivity of a fluid-saturated porous medium, with an insignificantly conductive matrix, depends nonlinearly on the porosity and the fluid electrical conductivity field $\sigma (\mathbf{x} )$ within the pore space (e.g., Torquato and Haslach 2002; Milton and Sawicki 2003). During DC-monitored saline tracer tests, the interstitial fluid electrical conductivity field $\sigma (\mathbf{x} )$ originates from the heterogeneous and time-dependent saline concentration field $c (\mathbf{x} )$, whereby $\sigma (\mathbf{x} )$ and $c (\mathbf{x} )$ are related by a monotonically increasing relationship (e.g., Sen and Goode 1992).

Time-lapse DC data have been routinely acquired during conservative saline tracer tests to retrieve solute transport parameters. Examples include the calibration of advective-dispersive transport models (e.g., Kemna et al. 2002; Vanderborght et al. 2005; Koestel et al. 2008) highlighting the ability to constrain the spreading dynamics of solutes, which in turn have a strong impact on their mixing rate (e.g., Dentz et al. 2011; Villermaux 2019). They have also been used to calibrate dual-domain transport models ( Singha et al. 2007; Day-Lewis et al. 2017), thereby, highlighting the sensitivity of DC conductivity to incomplete mixing. Regardless of context, there is always an implicit or explicit reliance on a petrophysical relationship linking the salinity field to the bulk electrical conductivity at some scale. Almost all petrophysical relationships (e.g., Archie’s law Archie 1942) ignore heterogeneity in fluid conductivity below the scale at which the petrophysical relationship is applied. This not only leads to biased estimates of mean saline concentrations (see the well-documented problem of apparent mass loss in, for instance, Singha and Gorelick 2005), but it also neglects potentially valuable information pertaining to mixing and spreading. Indeed, recent numerical modelling has demonstrated that bulk electrical conductivity time-series can constrain geostatistical models of log-hydraulic conductivity fields Visentini et al. (2020).

Advancing our understanding of how tracer heterogeneity affects bulk electrical conductivity is necessary to derive quantitative and robust spreading and mixing-related measures of the salinity field from geoelectrical data. Theoretical work on upscaling is needed, but also well-controlled experiments with simultaneous monitoring of upscaled electrical conductivities of porous medium samples together with highly-resolved imaging of concentration fields. Optically monitored tracer tests (e.g., Willingham et al. 2008; Anna et al. 2014; Jiménez-Martínez et al. 2015, 2017) with geoelectrical monitoring (e.g., Kozlov et al. 2012; Jougnot et al. 2018; Izumoto et al. 2020 provide a suitable framework for such experiments. For example, Kozlov et al. (2012) used a micro-model sample filled with brine, oil and air to highlight the percolation-driven response of bulk electrical conductivity. Another study Jougnot et al. (2018) considered advectively-dominated solute transport in saturated and unsaturated conditions to demonstrate that the apparent loss of mass that commonly plagues hydrogeophysical investigations is, at least partly, a consequence of assuming complete mixing (i.e., a constant salinity) below the averaging scale.

Milli-fluidic experiments combining optical- and electrical monitoring are challenging and few such studies have been performed to date. Errors in electrical monitoring and modelling, in the inferred time-evolving concentration field or in the concentration-conductivity relationship may lead to misleading findings. The strong connectivity-dependence of the salinity field on the electrical response places higher demands on the concentration imaging than in purely hydrological experiments (e.g., Willingham et al. 2008; Anna et al. 2014; Jiménez-Martínez et al. 2015, 2017). Consequently, there is a need for comparatively simple experiments covering well-known processes that can be used to ensure consistency in all aspects of the experimental setup, processing and modelling.

Here, we investigate the impact of diffusion-limited solute mixing on the temporal evolution of bulk electrical conductivity in a quasi two-dimensional (2-D) saturated porous medium. To this end, we have developed a milli-fluidic cell to monitor the transport of a saline and fluorescent tracer both optically and electrically. High resolution optical monitoring of the time-evolving 2-D (depth-averaged) solute concentration field is achieved by a fluorimetric technique combined with careful calibration to link light intensity with solute concentration. The resulting time-series of 2D concentration fields are used to predict the associated spatial distribution of local electrical conductivities of the fluid. These are then used to predict the apparent conductivities under various configurations of current injection and voltage measurements. Direct electrical measurements of apparent conductivities are also carried out, by equipping the flow cell with a resistivity meter. The porous medium consists of three regions with two contrasting permeabilities, one region of higher permeability being sandwiched across the medium’s width between two identical regions of lower permeability. We investigate the electrical signature of diffusive mass transfer from the high permeability region to the others.

The article is organized as follows. In Sect. 2, the materials and methods used for performing the experiment are described, including the tracer test procedure, the milli-fluidic, geoelectrical and optical acquisition setups, along with the fluorimetry technique and the associated image processing workflow. In Sect. 3, we provide a summary of the modelling approaches used to simulate DC-conductivity and solute diffusion. In Sects. 4 and 5, the main results are presented and discussed, respectively. Section 6 concludes the paper.

## Materials and Experimental Methods

### Porous Medium Design

**Fig. 1:**
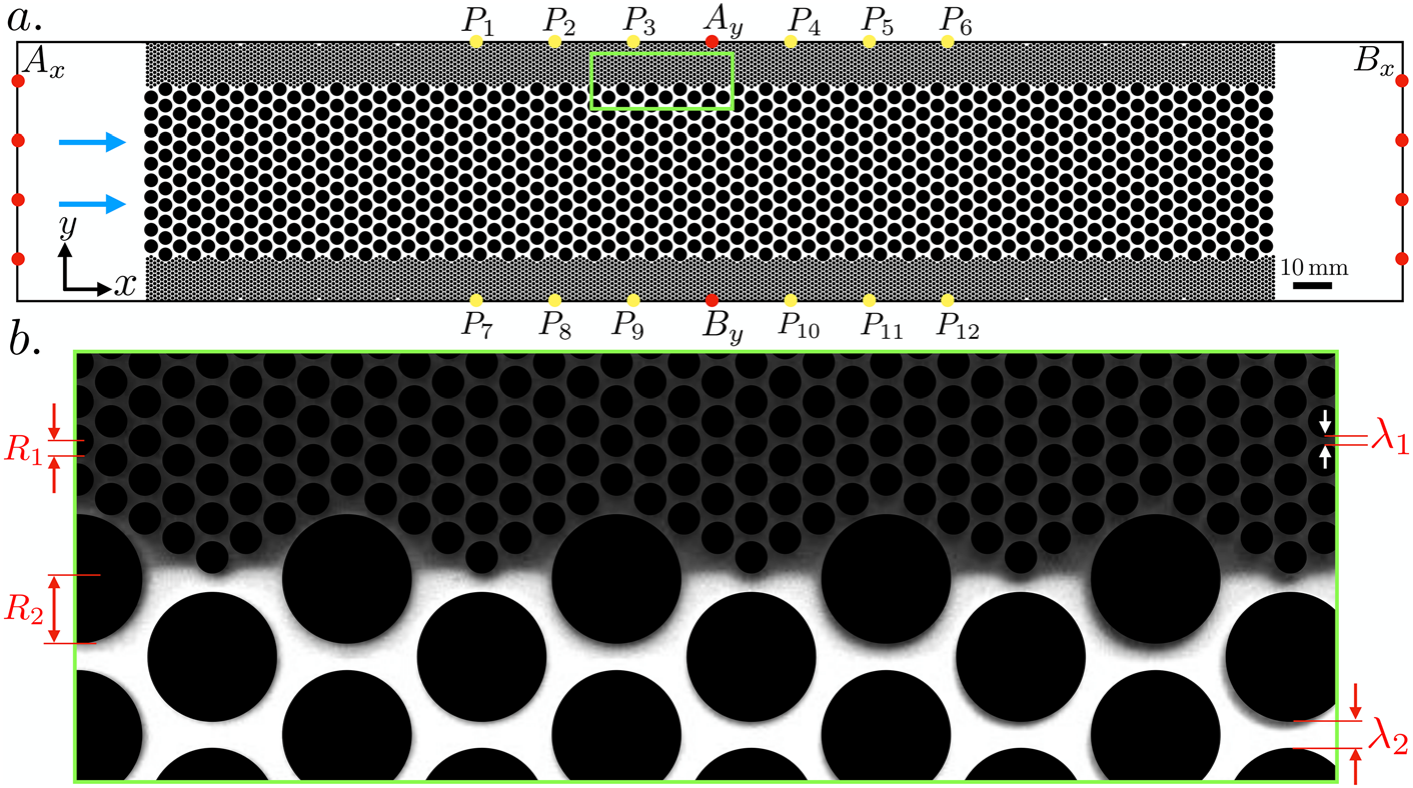
**a** Top schematic view of the flow cell including, from left to right, an inlet chamber, an artificial porous medium, and an outlet chamber. The blue arrows indicate the flow direction during tracer injection. The sets of current injection electrodes $A_x$-$B_x$ (measurement mode $\hbox {M}_x$) and $A_y$-$B_y$ (measurement mode $\hbox {M}_y$) are indicated with red dots and the potential electrodes with yellow dots. **b** Zoom-in of the region indicated by a green rectangle in (a) ($x \in \left[ 160,200\right]$ and $y \in \left[ 52,72\right] \,\hbox {mm}$) showing the interface between the top and the middle channels, the former saturated with the background solution (grey-colored) and the latter with fluorescent the tracer (white-colored), just after stopping the tracer injection. The radii of the grains and the length of the pore throats are indicated, respectively, as $R_1$ and $\lambda _1$, for the top and bottom channel, and as $R_2$ and $\lambda _2$, for the middle channel

We consider a polydimethylsiloxane (PDMS) flow cell of length $L = 590 \,\hbox {mm}$, width $w = 74 \,\hbox {mm}$ and height $h = 0.4 \,\hbox {mm}$ that contains a hexagonal lattice (of length $L_{\text {PM}} = 330 \,\hbox {mm}$) of impermeable cylindrical pillars, representing the grains of a porous medium (black disks in Fig. 1a). We designed the (quasi 2-D) porous system as three regular networks of pillars, designated from now on by top, middle and bottom channels. As indicated in Fig. 1b, the top and bottom channels are characterized by a grain radius $R_1 = 0.50 \,\hbox {mm}$ and pore throats of width $\lambda _1 = 0.175 \,\hbox {mm}$, while the middle one has $R_2 = 2 \,\hbox {mm}$ and $\lambda _2 = 0.70 \,\hbox {mm}$. The porosity is $\phi = 0.35$ and the pore volume is $3.42 \,\hbox {ml}$. For flow regimes characterized by low Reynolds number ($Re = \lambda \, v / \nu$, *v* being the average fluid velocity and $\nu$ its kinematic viscosity), the fluid motion is controlled by the confinement scale $\lambda$, as described by the Kozeny-Carman formula (e.g., Bear 1972): each channel permeability is proportional to $\lambda ^2$, thus, the permeability contrast between the channels is 16. This induces a temporal scale separation of the same amount (16) in the advective transport for the middle channel compared to the top and bottom channels. Details on the cell construction are given in Appendix A. Table 1 includes a summary description of the parameters used to describe the flow cell and porous medium geometry.

### Tracer Solutions

We used a water-soluble tracer that is optically detectable and electrically conductive. We prepared two mixtures of distilled water containing a fluorescent tracer (FS), fluorescein sodium salt (Sigma-Aldrich), and sodium chloride (NaCl). Solution 1 has FS and NaCl concentrations of 0.001 and $0.015 \,\hbox {g/l}$, respectively, and is used to saturate the cell before injecting the tracer Solution 2, which has FS and NaCl concentrations of 0.01 and $0.15 \,\hbox {g/l}$. Solution 1 is obtained by diluting Solution 2 by a factor of 15.

The FS concentration is measured with the optical method described in Sect. 2.5. It serves as a proxy for the concentration of NaCl, which is used to create a sufficiently large contrast between the electrical conductivities of the background and tracer mixtures (e.g., Jougnot et al. 2018). The FS molecule is about 3 times larger than the NaCl molecule, which results in a factor of $\sim 3.8$ between the diffusion coefficients of the salts, with $D_{\text {FS}} \sim 0.42 \times 10^{-9} \,{\hbox {m}^2\hbox { s}^{-1}}$ and $D_{\text {NaCl}} \sim 1.6 \times 10^{-9}\,{\hbox {m}^2\hbox { s}^{-1}}$ (e.g., Casalini et al. 2011). The lower mobility of FS and the considered 1:15 concentration ratio imply that this tracer contributes $\sim 57$ times less to the fluid’s electrical conductivity than the NaCl (e.g., Lesmes and Friedman 2005).

Injection of the heavier Solution 2 could lead to some gravity-induced stratification. However, the small cell thickness is likely to prevent the development of a pronounced vertical concentration profile. Moreover, given that our measurements of bulk electrical conductivity utilize electrodes that traverse the cell’s height entirely (see Sect. 2.4), tracer mass (fluid conductivity) that is segregated into parallel layers is averaged arithmetically, which leaves the bulk conductivity of the sample insensitive to any stratification.

**Table 1:** List of key symbols and parameters used throughout the manuscript

Symbols and parameter values	Description
*Porous medium*	
$L = 590$, $w = 74$ and $h = 0.4$. $L_{PM} = 330$ ($\hbox {mm}$)	Length, width and height of the flow cell. Length of the porous medium
$R_1 = 0.50$ and $R_2 = 2.00$. $\lambda _1 = 0.175$ and $\lambda _2 = 0.70$ ($\hbox {mm}$)	Cylindrical pillars radii corresponding to the top and bottom, and middle networks, respectively. Corresponding pore throat lengths
$w_{\text {ch}} = 12$. $w_{\text {middle}} = 50$ ($\hbox {mm}$)	Top and bottom low-permeability channel widths. Middle high-permeability channel width
*Tracer solutions*	
$D_{\text {FS}} \sim 0.42 \times 10^{-9}$ and $D_{\text {NaCl}} \sim 1.6 \times 10^{-9}$ (${\hbox {m}^2\hbox { s}^{-1}}$)	Diffusion coefficients of fluorescein and sodium chloride, the two used tracers
$\sigma _{\text {S}_1} = 0.0218$ and $\sigma _{\text {S}_2} = 0.2728$ (${\hbox {S m}^{-1}}$)	Electrical conductivity of Solution 1 and Solution 2, used for injection and background, respectively
*Electrical monitoring*	
$\{A_{x}, B_{x}\}$ and $\{A_{y}, B_{y}\}$	Current electrode sets for the *x* (longitudinal) and *y* (transversal) measurement modes, respectively, $\text {M}_x$ and $\text {M}_y$
*Time parameters*	
$t^{\text {NaCl}}_{\text {ch}} = 127300$ and $\tau _v = 33$ ($\hbox {s}$)	Diffusive (NaCl) and advective transport time-scales associated with, respectively, $w_{\text {ch}}$ and $L_{\text {PM}}$
$t^{\text {NaCl}}$	Temporal coordinate used to present the results, defined in terms of $t^{\text {NaCl}}_{\text {ch}}$ and $\tau _v$

### Flow System

Solution 2 is injected from a $60 \,\hbox {ml}$ syringe that is pushed by a syringe pump (Harvard Apparatus PHD 22/2000 Series). To ensure full saturation within the flow cell, it is initially lifted from the outlet side and fixed at a nearly vertical position with the inlet side at the bottom. The air is pushed out by slowly injecting (heavier) carbon dioxide (CO$_2$) from the inlet during approximately one hour. After flushing with CO$_2$, 52 pore volumes of Solution 1 are slowly injected within a period of $48 \,\hbox {hours}$ to allow the trapped CO$_2$ bubbles to diffuse into the liquid and flush away. Once the flow cell is fully saturated with Solution 1, the experiment starts at $t = 0 \,\hbox {s}$ and Solution 2 is injected at a volumetric flow rate $Q = 0.1 \,{\hbox {ml s}^{-1}}$ resulting in a mean advective velocity of $v \sim 10 \,{\hbox {mm s}^{-1}}$, until $t_{\text {stop}} = 60 \,\hbox {s}$. This injection inverval was chosen to saturate the middle more permeable porous channel with the injected tracer, while leaving the top and bottom channels predominantly filled with Solution 1 (see Fig. 1b). After stopping the injection, the tracer is left to diffuse through the cell for 4 days. During the experiment, a full set of electrical measurements are made every $24 \,\hbox {s}$, while the FS concentration is measured optically, as described in Sect. 2.5.

### Geoelectrical Monitoring System

Since we expect electrical anisotropy at the sample scale, we measure the apparent electrical conductivity both along the *x*- and *y*-directions. To do so, we inject a voltage square wave of $\varDelta V = 1 \,\hbox {V}$ amplitude and $12 \,\hbox {s}$ period (see Appendix B for details) every $12 \,\hbox {s}$, alternatively either through the sets of injection electrodes $A_x$-$B_x$ or through the set $A_y$-$B_y$ (see Fig. 1a). These two injection modes are denoted $\hbox {M}_{x}$ and $\hbox {M}_{y}$, respectively, and we collect measurements of: (i) the electric currents $i_x$ and $i_y$ flowing between either set of injection electrodes, stored as $I_x(t)$ and $I_y(t)$, and (ii) the resulting voltages between each of the eleven potential electrodes $P_i$ ($i = 2,\ldots ,12$) and the reference electrode $P_1$. These potential electrodes are distributed along the boundary of the porous medium (see Fig. 1a). Invoking the superposition principle, the voltage between any two electrodes $P_i$ and $P_j$ is obtained by subtracting the voltage measured between $P_j$ and $P_1$ from the one measured between $P_i$ and $P_1$. This allows us to construct 66 voltage-difference time-series for each injection mode, collectively grouped as the columns of the corresponding arrays $\mathbf{V} _x(t)$ and $\mathbf{V} _y(t)$. Finally, the resistance time-series corresponding to M$_{x}$ and M$_{y}$ are obtained as $\mathbf{R} _x(t) = \mathbf{V} _x(t)/I_x(t)$ and $\mathbf{R} _y(t) = \mathbf{V} _y(t)/I_y(t)$, respectively, and contain data sampled every $24 \,\hbox {s}$.

The current injection and measurement protocols are interfaced with the datalogger Campbell Micrologger CR3000, which executes all operations. To establish electrical contacts with the cell, we use stainless steel cylindrical electrodes of $1 \,\hbox {mm}$ diameter that are inserted through the PDMS such that they are in contact with the fluid at the designated locations.

### Image Acquisition

The concentration field of FS is measured optically from the intensity of its emitted light at wavelengths around $\lambda _{\text {em}} = 521 \,\hbox {nm}$, when excited by a light source at $\lambda _{\text {ex}} = 494 \,\hbox {nm}$ (e.g., Anna et al. 2014; Jiménez-Martínez et al. 2015, 2017; Jougnot et al. 2018; Sjöback et al. 1995). To reduce tracer bleaching occurring during FS exposure to light (e.g., Imamura and Koizumi 1955), we use a flash lamp, placed about $60 \,\hbox {cm}$ above the cell, that is activated by an electronic signal (transistor–transistor logic gate, TTL). The latter is triggered from a computer via the open-source software Micromanager, used also to control a CCD camera (Ximea, MD120MU-SY), placed about $85 \,\hbox {cm}$ below the cell. The flash lamp is combined with a parabolic umbrella with a reflective internal surface focusing the emitted light towards the flow cell and the camera below it. Moreover, a translucid white sheet nylon covering the open side of the umbrella helps to homogenize the light over the flow cell. The experiment takes place in a darkroom to avoid any light disturbance. The camera exposure time is set to $0.4 \,\hbox {s}$: as soon a picture acquisition is triggered, the controlling software triggers the flash that lasts for a few tens of $\hbox {ms}$. A band pass optical filter (LEE 126 Mauve) is placed between the flash lamp and the flow cell to illuminate it with light having wavelengths below $500 \,\hbox {nm}$. A band pass optical filter (Edmund Optics $520 \,\hbox {nm}$ CWL, $10 \,\hbox {nm}$ Bandwidth) is placed in front of the camera to only allow light in the window $[510{-}530] \,\hbox {nm}$ to reach its sensor.

The camera records gray-scale light intensity images of $2832 \times 4244$ pixels with intensities ranging from 0 to 255 (8-bit pixel depth camera). The length of a pixel corresponds to $0.06 \,\hbox {mm}$, which implies that the pore throats of the finely-grained porous channels are resolved with 3 pixels. The collected time-series of light intensity images, *I*(*x*, *y*, *t*), consist of 2334 pictures sampled as follows: (i) the first 90 every $2 \,\hbox {s}$, then (ii) 490 every $60 \,\hbox {s}$ and then (iii) 1889 every $600 \,\hbox {s}$. This sampling scheme follows the dynamics of our tracer test: fastest at the beginning and slower with time.

Note that in the following *x*-, *y*- and *t* interchangeably denote discrete spatial and temporal indices, used for describing the experiment, or continuous coordinates. We prefer such a loose notation for reasons of simplicity and because their meaning is facilitated by context.

### Image Processing

To obtain concentration images, we first build a binary image, a so-called pore space mask, containing the spatial distribution of the pore space and grains in terms of a 2-D indicator function that takes values of 1 inside the pores and 0 elsewhere (see Appendix C for details on its compilation). We then transform *I*(*x*, *y*, *t*) into time-series of FS concentration fields, *c*(*x*, *y*, *t*) using a calibrated relationship. To establish this relationship, we collected a series of 6 pictures $I^{i}_c(x,y)$ ($i = 1,\ldots ,6$) of the flow cell when it was uniformly saturated with 6 corresponding solutions having FS concentrations $C^{i}$ ($i = 1,\ldots ,6$) of 0.00100 (corresponding to Solution 1), 0.00195, 0.00300, 0.00500, 0.00833 and $0.01500 \,\hbox {g/l}$ (corresponding to Solution 2). These solutions are obtained by successive dilution of Solution 2 until Solution 1 is obtained. The concentrations follow approximately a log-equidistant distribution and are slowly injected over successive periods of $\sim 36 \,\hbox {h}$ in an increasing order of concentration, using 52 pore volumes for each of them to ensure uniform concentration fields. After, both $I^{i}_c(x,y)$ and *I*(*x*, *y*, *t*) are registered (e.g., Brown 1992) using the grain mask as reference, in order to ensure that (*x*, *y*) represent the same position within the flow cell for any image. Such a procedure, combined with the fact that both the camera and flow cell are fixed to an aluminum solid structure during the whole duration of the experiment and calibration, allows us to employ a pixel-by-pixel transform of the acquired light intensity *I*(*x*, *y*, *t*) into the concentration field *c*(*x*, *y*, *t*). We use a local Piecewise Cubic Hermite Interpolating Polynomial on the calibration data $I^{i}_c(x,y)$-$C^{i}$.

### Concentration: Electrical Conductivity Calibration

We calibrate an empirical relationship between concentration and fluid electrical conductivity, in order to transform *c*(*x*, *y*, *t*) into the electrical conductivity field $\sigma (x,y,t)$. To do so, we rely on electrical conductivity measurements of the calibration solutions $C^{i}$, using the *WTW ProfiLine Cond 3310* portable conductivity meter. This yields conductivity values of $\sigma _{\text {S}_1} = 0.0218$ and $\sigma _{\text {S}_2} = 0.2728 \,{\hbox {S m}^{-1}}$ for Solution 1 and 2, respectively, giving $\sigma _{\text {S}_2}/\sigma _{\text {S}_1} = 12.51$.

## Modelling

### Electrical Modelling

Once the time-series $\sigma (x,y,t)$ have been obtained, they are used as inputs to the governing Laplace equation

1 $$\begin{aligned} \varvec{\nabla }\cdot (\sigma \varvec{\nabla }\phi ) = 0 , \end{aligned}$$

where $\phi$ is the electrical potential. Equation 1 is solved with the finite-element solver module of the open-source Python library pyGIMLi (Rücker et al. 2017), using mixed Dirichlet–Neumann boundary conditions corresponding to the two injection modes. Based on conductivity measurements performed on the PDMS material, the grains are assigned a conductivity of $10^{-9} \,{\hbox {S m}^{-1}}$. Additional simulations (not shown) considered values ranging between $10^{-6} \,{\hbox {S m}^{-1}}$ and $10^{-10} \,{\hbox {S m}^{-1}}$ without any noticeable influence on the results. For $\hbox {M}_{x}$, values of 1 and $0 \,\hbox {V}$ are imposed for $\phi$, respectively, at the positions of the current electrodes $A_x$ (left) and $B_x$ (right) (Fig. 1), respectively, and no-flux conditions are imposed on the remaining boundaries. For $\hbox {M}_{y}$, values of 1 and $0 \,\hbox {V}$ are imposed, respectively, at the positions of $A_y$ and $B_y$, and a no-flux condition is imposed on the remainder of the cell’s perimeter. This yields a pair of time-series of electrical potential fields $\phi _{\text {M}_x}(x,y,t)$ and $\phi _{\text {M}_y}(x,y,t)$, corresponding to each injection mode, sampled at the positions of the potential electrodes $P_i$ ($i = 1,\ldots ,12$) from which 66 voltage time-series are obtained (see Sect. 2.4). They are grouped into the arrays $\mathbf{V} _x^\text {sim}(t)$ and $\mathbf{V} _y^\text {sim}(t)$. From the corresponding simulated time-series of electrical current, $\mathbf{I} _x^\text {sim}(t)$ and $\mathbf{I} _y^\text {sim}(t)$, the simulated resistance time-series are obtained as $\mathbf{R} _x^\text {sim}(t) = \mathbf{V} _x^\text {sim}(t)/\mathbf{I} _x^\text {sim}(t)$ and $\mathbf{R} _y^\text {sim}(t) = \mathbf{V} _y^\text {sim}(t)/\mathbf{I} _y^\text {sim}(t)$.

The camera images *I*(*x*, *y*, *t*) and, hence, the resulting concentration estimates *c*(*x*, *y*, *t*) include neither the inlet nor the outlet chambers. Three modeling scenarios including these chambers were used to assess their influence on the electrical resistance time-series. First, we assumed the chambers to be filled with Solution 1 (background conductivity). Second, we considered the chambers to be filled with Solution 2 (injected tracer conductivity). Third, we assumed that the inlet chamber was filled with Solution 2 and the outlet chamber with Solution 1. Considering both measurement modes $\hbox {M}_{x}$ and $\hbox {M}_{y}$, the observed discrepancies among these scenarios were below $0.8 \%$.

### Post-processing of Electrical Resistance Time-Series

The observed (simulated) electrical resistance time-series $\mathbf{R} _x(t)$ and $\mathbf{R} _y(t)$ ($\mathbf{R} _x^\text {sim}(t)$ and $\mathbf{R} _y^\text {sim}(t)$) are transformed into corresponding time-series of apparent conductivities $\Sigma _{\text {M}_x} (t)$ and $\Sigma _{\text {M}_y} (t)$ ($\Sigma _{\text {M}_x} ^\text {sim}(t)$ and $\Sigma _{\text {M}_y} ^\text {sim}(t)$) by, first, multiplying the columns of the resistance arrays with the corresponding 66 geometrical factors, that account for the measurement and sample geometry in a homogeneous medium (hence the term “apparent”) (e.g., Keller and Frischknecht 1966), and, second, inverting element-wise the resulting arrays. The geometrical factors are obtained by numerical simulations following a procedure similar to that of Jougnot et al. (2010). Namely, the resistance is simulated using a domain of the same dimensions as the experimental cell but of unit porosity (i.e., without grains), which is saturated with a known value of fluid resistivity. Since the apparent resistivity should equate the input fluid resistivity, the corresponding geometrical factor is obtained by dividing the latter by the simulated resistance. This process is repeated for each measurement configuration.

The simulated time-series $\Sigma _{\text {M}_x} ^\text {sim}(t)$ and $\Sigma _{\text {M}_y} ^\text {sim}(t)$ are calculated on a 2-D geometry that implicitly assumes a $1 \,\hbox {m}$-thick third dimension. Consequently, they are multiplied by the ratio between the cell’s nominal thickness and $1 \,\hbox {m}$, that is, $4 \cdot 10^{-4}$, in order to make them comparable with the measured time-series $\Sigma _{\text {M}_x} (t)$ and $\Sigma _{\text {M}_y} (t)$.

**Fig. 2:**
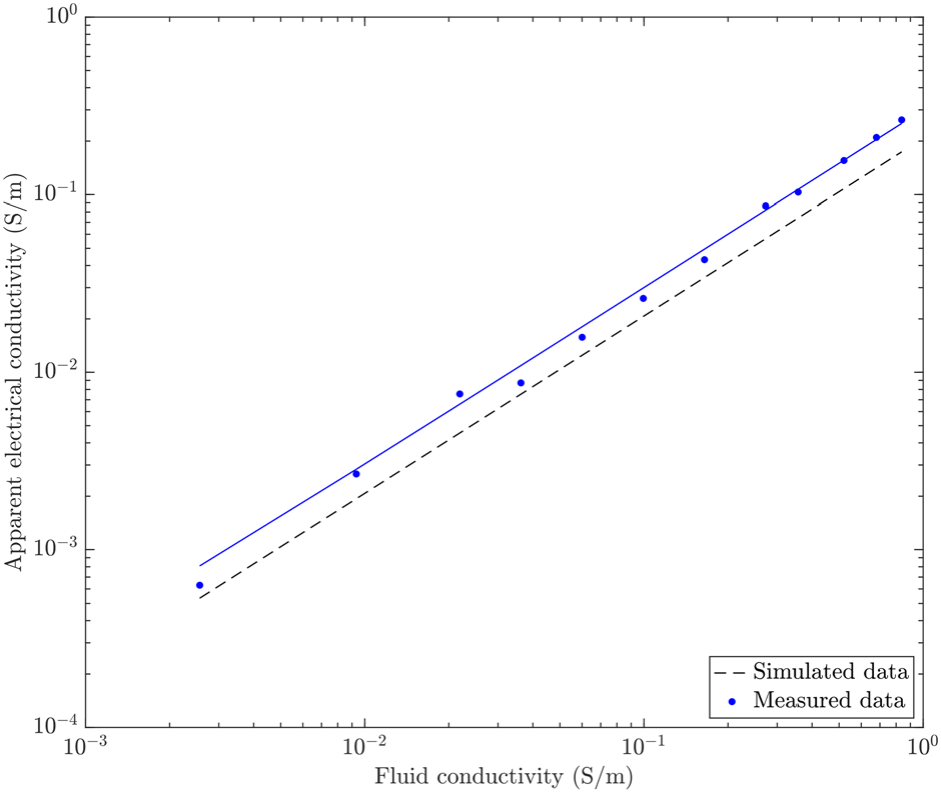
Fluid electrical conductivity versus apparent electrical conductivity for a homogeneous solute concentration. The measured apparent electrical conductivities are shown as blue dots with the associated regression as a continuous black line. The numerically calculated apparent electrical conductivities are shown as a black dashed line

We now consider the bulk electrical conductivity (Fig. 2) calculated using the potential electrode pair $P_1$-$P_6$ and the horizontal injection mode M$_{x}$ for 12 different solute concentrations (including those of Solution 1 and 2). The data plot on a linear log-log plot and extrapolation to zero fluid conductivity suggests a negligibly small bulk electrical conductivity. This confirms that the PDMS pillars are electrical insulators and that all electrical conduction takes place in the liquid phase (i.e., no measurable surface conductivity). Thus, we can use Archie’s law Archie (1942) to relate the electrical conductivity of the homogeneous interstitial fluid, $\sigma _\text {fluid}$, to that of the porous medium, $\sigma _{\text {PM}}$, by

2 $$\begin{aligned} \sigma _{\text {PM}} = \frac{\sigma _\text {fluid}}{F}, \end{aligned}$$

with *F* the formation factor that is the same in all channels within the artificial porous medium. Indeed, the pore geometries in the low and high permeability channels differ only by a (size) scaling factor so their porosities and topology are identical by design. The regression line has a slope of 1/3.34, which yields an empirical formation factor $F_{\text {data}} = 3.34$. The corresponding simulated values of apparent conductivity (i.e., using $P_1$-$P_6$), plot slightly differently (see dashed line in Fig. 2), yielding a theoretical formation factor $F_{\text {sim}} = 4.82$.

In order to ensure that $F_\text {data} = F_\text {sim}$, an effective cell thickness of $0.57 \,\hbox {mm}$ is finally used (instead of the cell’s nominal thickness of $0.4 \,\hbox {mm}$) when normalizing the simulated data. This slightly larger cell thickness is likely due to small errors occurring during the porous system fabrication (see Appendix A). Indeed, too deep cylindrical pillars could result from an imperfect drilling operation when fabricating the stainless steel mould. Also, some slight thickness variations (increments) may have arisen if the PDMS plate was not sandwiched perfectly flat between the acrylic plates, that is, with a slight topography.

### Solute Diffusion Modelling in Confined Media

In order to model diffusion within our experimental cell after tracer injection, we adopt a Eulerian standpoint and consider the diffusion equation in both 1-D and 2-D. The 1-D model is used as a comparison tool against our observed concentration profiles (see Fig. 4), whereas the 2-D model is used in the context of the numerical investigation in Sect. 5.2.1.

For 1-D diffusion, we consider a confined domain of length $L = \infty$ and width $w = 74 \,\hbox {mm}$, subjected to homogeneous Neumann (i.e., impermeable walls) boundary conditions. Prescribing an initial condition $f({\hat{y}})$ (with ${\hat{y}} = y - w/2$), that is, a *y*-oriented concentration profile at the end of the injection, the corresponding boundary-value problem admits the following closed-form solution in terms of a Fourier cosine representation (e.g., Balluffi et al. 2005):

3 $$\begin{aligned} c({\hat{y}},t) = \sum ^\infty _{n=0} b_n \cos { (\pi n {\hat{y}}) } \exp { \Big \{\frac{-n^2 \pi ^2 D t}{w^2} \Big \} }, \end{aligned}$$

where *D* is the diffusion coefficient and the weights $b_n$ are the Fourier coefficients of the series, given by

4 $$\begin{aligned} b_n = \frac{2}{L} \int _0^L f({\hat{y}}') \cos { (\pi n {\hat{y}}) } d{\hat{y}}'. \end{aligned}$$

For 2-D diffusion, we consider a confined domain of length $L = 590 \,\hbox {mm}$ and width $w = 74 \,\hbox {mm}$ (as in the experimental cell, Sect. 2.1), with a no-flux condition along all the boundaries. Different initial conditions for the modelled NaCl concentration field are considered (Sect. 5.2.1) and the resulting boundary-value problem is numerically solved using the finite difference implementation included within the groundwater solute transport simulator package MT3D-USGS Bedekar et al. (2016).

#### Characteristic Transport Time-Scales Within the Cell

We consider characteristic diffusive $\tau _{\text {ch}}^{\text {NaCl}}$ and advective $\tau _v$ transport time-scales for the NaCl associated with, respectively, the length scales (i) $w_\text {ch} = 12 \,\hbox {mm}$ corresponding to the width of the top and bottom low-permeability channels and (ii) $L_{\text {PM}}$ corresponding the porous medium *x*-length. These are defined as $\tau _{\text {ch}}^{\text {NaCl}} = w_\text {ch}^2 /D^\text {eff}_{\text {NaCl}} \sim 127\,300 \,\hbox {s}$ ($\sim 16 \,\hbox {h}$) and $\tau _v = L_{\text {PM}} / v \sim 33 \,\hbox {s}$. Note that we have replaced the bulk diffusion coefficient $D_{\text {NaCl}}$ by the effective diffusion coefficient $D_{\text {NaCl}}^\text {eff} = D_{\text {NaCl}} / \sqrt{2}$ to account for the tortuosity of the porous medium (e.g., Bear 1972). The smaller effective rate of diffusion is a consequence of the solute needing to travel a longer path in order to circumvent the grains.

#### Time Handling

Since the measured apparent electrical conductivity time-series $\Sigma _{\text {M}_x}(t)$ and $\Sigma _{\text {M}_y}(t)$ are predominantly sensitive to the NaCl concentration field, we present such data as a function of $t^{\text {NaCl}}$, a temporal coordinate defined as a function of *t* as:

5 $$\begin{aligned} t^{\text {NaCl}} = {\left\{ \begin{array}{ll} (t-t_{\text {stop}})/\tau _v &{} 0 \le t\le t_{\text {stop}} \\ (t-t_{\text {stop}})/\tau _\text {ch}^{\text {NaCl}} &{} t> t_{\text {stop}}, \end{array}\right. } \end{aligned}$$

where $t_{\text {stop}}$ refers to the end of the tracer injection (Sect. 2.3) and $\tau _v$ and $\tau _\text {ch}^{\text {NaCl}}$ have been defined in and Sect. 3.3.1. The origin is established at $t_{\text {stop}}$ as the main interest lies in the times after injection has stopped. With this convention, negative and positive $t^{\text {NaCl}}$ represent, respectively, times normalized by advective and diffusion transport time-scales. The FS sensed by the optical measurements diffuses $\sim 3.8$ times slower (Sect. 2.2) than the NaCl sensed by the DC data. Thus, to make geoelectrical and image-based outputs comparable, we will present the latter as a function of $t^{\text {NaCl}}$/3.8 for $t^{\text {NaCl}} > 0$. This implies that our 4-day experiment provides us with fluorimetry-inferred NaCl concentration fields over 4/3.8 days.

## Results

We now analyze the data obtained during the tracer test. First, we consider the fluorimetry-inferred NaCl concentration field (Sect. 3.1) using four snapshot examples. Then, we present measured apparent electrical conductivity time-series and compare them with simulations.

### Fluorimetry-Inferred NaCl Concentration Field

**Fig. 3:**
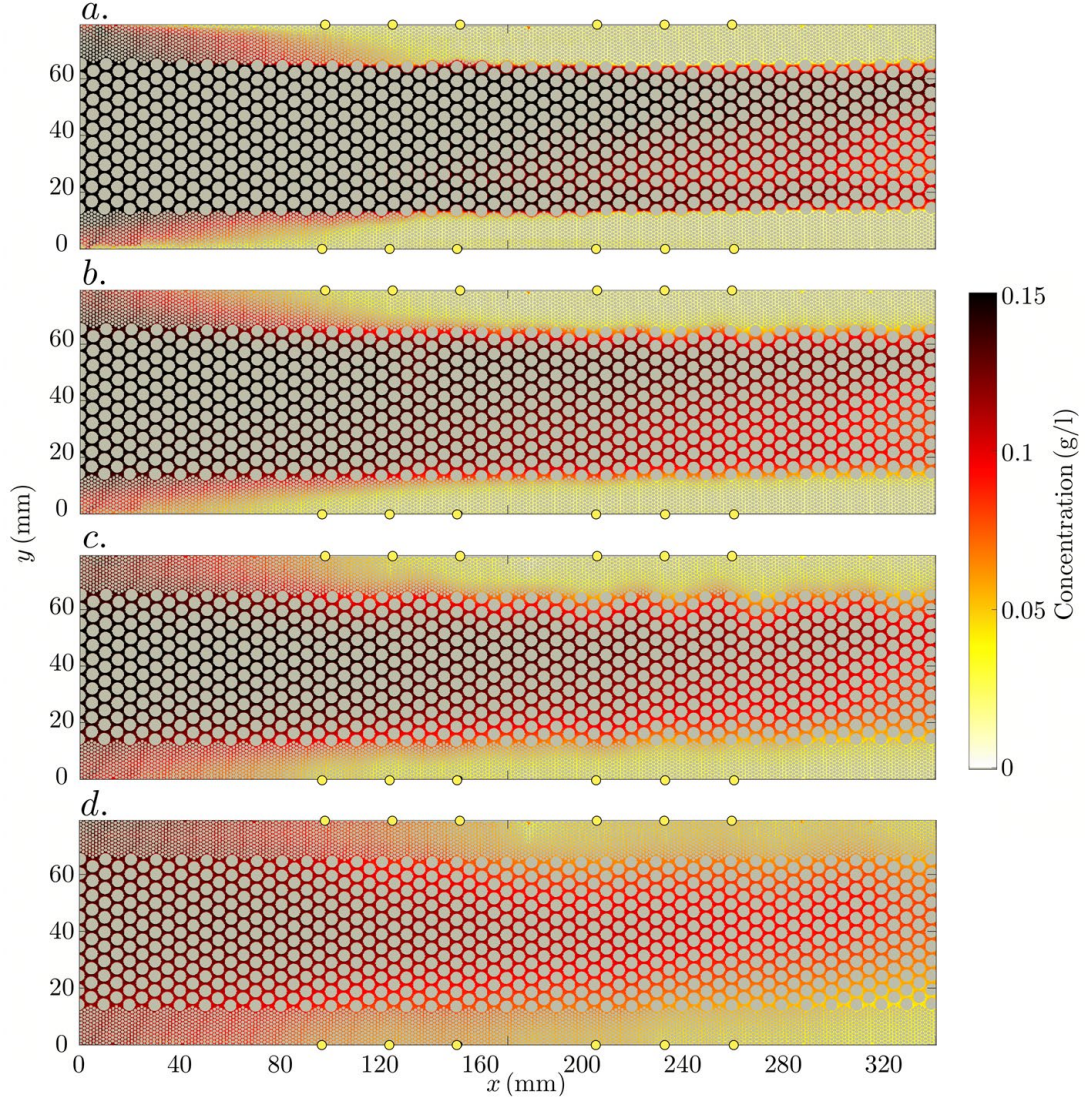
Four snapshots of the fluorimetry-inferred NaCl concentration fields corresponding to times (**a**) $t^{\text {NaCl}}_1 = 0$, (**b**) $t^{\text {NaCl}}_2 = {5\times 10^{-3}}$, (**c**) $t^{\text {NaCl}}_3 = {5\times 10^{-2}}$, and (**d**) $t^{\text {NaCl}}_4 = {5\times 10^{-1}}$. The yellow dots indicate the potential electrodes, see Fig. 1 for the naming convention

Snapshots of the fluorimetry-inferred NaCl concentration field are shown for the following $t^{\text {NaCl}}$ times (Sect. 3.3.2): $t^{\text {NaCl}}_1 = 0$ (Fig. 3a), that is, just after the tracer injection has been stopped; $t^{\text {NaCl}}_2 = {5\times 10^{-3}}$ (Fig. 3b) and $t^{\text {NaCl}}_3 = {5\times 10^{-2}}$ (Fig. 3c), approximate times at which the apparent electrical conductivity data show, respectively, an accelerated growth rate and a local maximum (Sect. 4.2); and $t^{\text {NaCl}}_4 = {5\times 10^{-1}}$ (Fig. 3d), that is, close to the last available fluorimetry-inferred NaCl concentration field.

For $t^{\text {NaCl}}_1$ and $x > 160 \,\hbox {mm}$ (Fig. 3a), there is a sharp concentration gradient along the *y*-direction, marking the boundaries between the permeability channels (see also the zoom in Fig. 1b). The high permeability middle channel does not exhibit a perfectly homogeneous concentration distribution as evidenced by lower values in the middle. We attribute this to slight thickness variations in the inlet chamber, also seen in Jougnot et al. (2018). For $x < 160 \,\hbox {mm}$, the tracer has slightly invaded the low permeability channels up to the *x*-locations of $P_2$ and $P_8$ (Fig. 1a). For $t^{\text {NaCl}}_2$ and $t^{\text {NaCl}}_3$ (Fig. 3b, c), the initially sharp concentration gradient along the *y*-direction is decreased. At the last considered time ($t^{\text {NaCl}}_4$, Fig. 3d), the NaCl concentration field has achieved a much higher degree of homogeneity in comparison with $t^{\text {NaCl}}_1$.

**Fig. 4:**
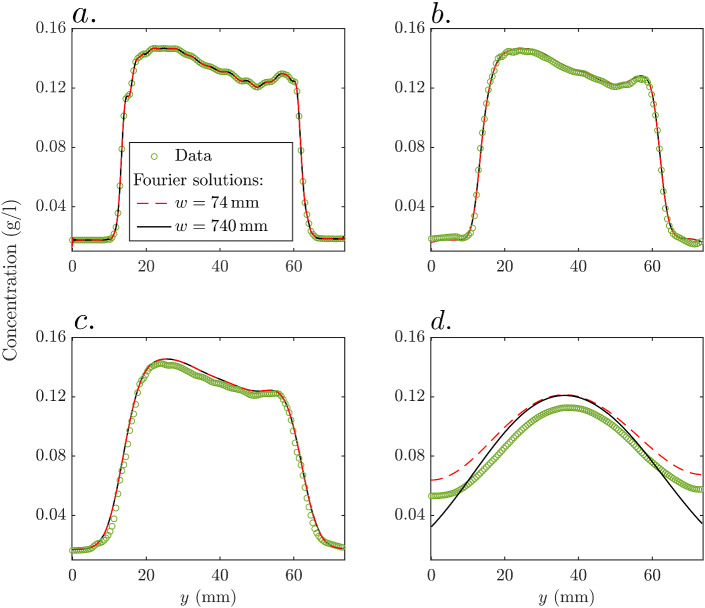
Four snapshots of fluorimetry-inferred and modeled NaCl concentration profiles for times **a** $t^{\text {NaCl}}_1 = 0$ (initial conditions for the modeling), **b** $t^{\text {NaCl}}_2 = {5\times 10^{-3}}$, **c** $t^{\text {NaCl}}_3 = {5\times 10^{-2}}$, and **d** $t^{\text {NaCl}}_4 = {5\times 10^{-1}}$

Snapshots of *y*-oriented fluorimetry-inferred NaCl mean concentration profiles for the times considered above are shown in Fig. 4. These are obtained by *x*-averaging the 2-D concentration field within the horizontal coordinate range $x \in \left[ 140, 175\right] \,\hbox {mm}$. Additionally, we present analytically computed NaCl concentration profiles (Eq. 3) assuming a 1D domain. This is done for the same width *w* as that of our porous medium, but also for a ten times larger width, to mimic an unconfined domain. As initial condition, we consider the first snapshot of the *x*-averaged concentration profile, resulting in a perfect match between the modelled and observed concentration profiles at $t^{\text {NaCl}}_1$.

The concentration profile at $t^{\text {NaCl}}_1$ (end of the injection) is not a perfect step function and, notably, it features a non-flat topography in its central part (Fig. 4a). This is caused by an imperfect tracer injection pattern, as mentioned previously when discussing Fig. 3. At $t^{\text {NaCl}}_2$ (Fig. 4b), a small degree of smoothing has occurred, along with a slight amplitude decay ($\sim 2 \%$) of the observed concentration profile with respect to the analytically calculated profiles. At $t^{\text {NaCl}}_3$ (Fig. 4c), the smoothing, as well as the magnitude decay of the observed data, is more pronounced (with an average of $\sim 4 \%$ within the central part), the latter indicating an ongoing loss of solute mass from the considered range $x \in \left[ 140, 175\right]$ which is not accounted for in the 1D solutions. At $t^{\text {NaCl}}_4$ (Fig. 4d), the NaCl profiles display a much higher degree of homogeneity compared with the situation at $t^{\text {NaCl}}_1$. Particularly, a considerable amount of NaCl mass has been brought by diffusion to the low permeability channels. The shapes of the observed and analytical solution for the confined domain (i.e., $w = 74 \,\hbox {mm}$) are very similar, although the magnitude of the former is $\sim 9 \%$ smaller than that of the latter. As expected, the analytical solution for the unconfined solution (i.e., $w = 740 \,\hbox {mm}$) underestimates concentrations in the vicinity of the boundaries.

**Fig. 5:**
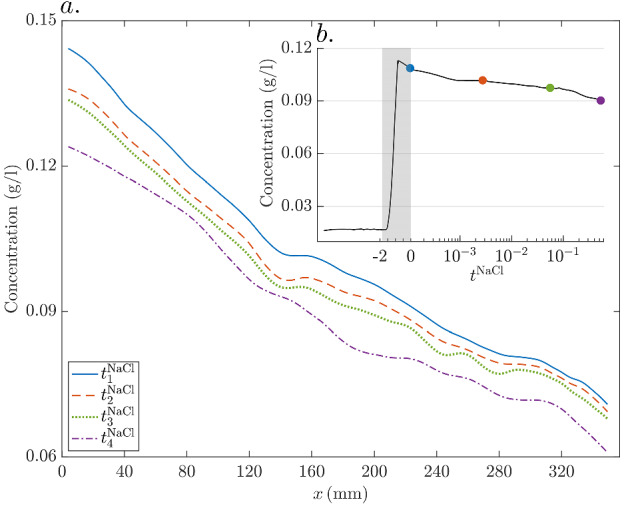
**a** *y*-averaged mean of the fluorimetry-inferred NaCl concentrations as a function of the horizontal coordinate *x* for times $t^{\text {NaCl}}_1 = 0$ (blue solid line), $t^{\text {NaCl}}_2 = {5\times 10^{-3}}$ (red-dashed line), $t^{\text {NaCl}}_3 = {5\times 10^{-2}}$ (green dotted line), and $t^{\text {NaCl}}_4 = {5\times 10^{-1}}$ (purple dotted-dashed line). **b** Temporal evolution of the inferred mean concentration over the entire field of view. Note that negative and positive $t^{\text {NaCl}}$ represent, respectively, times normalized by advective and diffusion transport time-scales (Sect. 3.3.2). The grey-colored rectangle highlights the tracer injection period. The blue, red, green and purple-colored dots on the curve indicate the mean concentration field values at times $t^{\text {NaCl}}_1$, $t^{\text {NaCl}}_2$, $t^{\text {NaCl}}_3$ and $t^{\text {NaCl}}_4$

In Fig. 5, *y*-averaged concentration profiles are plotted as a function of *x* for the same four times. The longitudinal profile at $t^{\text {NaCl}}_1$ (blue solid line in Fig. 5) shows a decreasing trend from left to right. The ratio of mean concentration in the inlet to the outlet is $\sim 1.8$. This is mainly due to the invasion of the tracer along the low permeability channels in the inlet region, and by the incomplete tracer saturation of the middle high permeability channel close to the outlet region (Fig. 3a). At $t^{\text {NaCl}}_2$ (red dashed line in Fig. 5), the ratio of inlet to outlet concentrations has been slightly reduced to $\sim 1.75$, suggesting that the mass imbalance along the *x*-direction is being mitigated by the action of *x*-directed mass transport by molecular diffusion. At the subsequent times $t^{\text {NaCl}}_3$ and $t^{\text {NaCl}}_4$ (respectively, green dotted and purple dotted-dashed lines in Fig. 5), the ratios decrease further to 1.7 and 1.5, respectively. Note also that the curves in Fig. 5 as a whole exhibit a downward shift with time, indicating that the total FS mass within the field of view, from which the NaCl concentrations are derived, has decreased either due to photo-bleaching or horizontal mass transport. The mean concentration, calculated by integrating the concentration field over the entire field of view, decreases throughout the experiment from 0.11 to $0.09 \,\hbox {g/l}$ (inset in Fig. 5), suggesting an apparent loss of tracer mass. This issue is discussed in Sect. 5.1.

### Apparent Electrical Conductivity Time-Series

We consider three potential electrode pairs having different relative orientations with respect to the predominant direction of diffusive mass transport (i.e., the *y*-direction). The chosen pairs are located close to the cell’s center with respect to the *x*-direction in order to have a minimal influence from the inlet and outlet chambers. The electrode pair $P_3$-$P_9$ measures a voltage in a direction that is aligned with the *y*-direction, $P_4$-$P_5$ in a direction aligned with the *x*-direction and $P_4$-$P_9$ along a diagonal line that crosses the cell’s center (see Fig. 1a for details concerning the electrode positions).

**Fig. 6:**
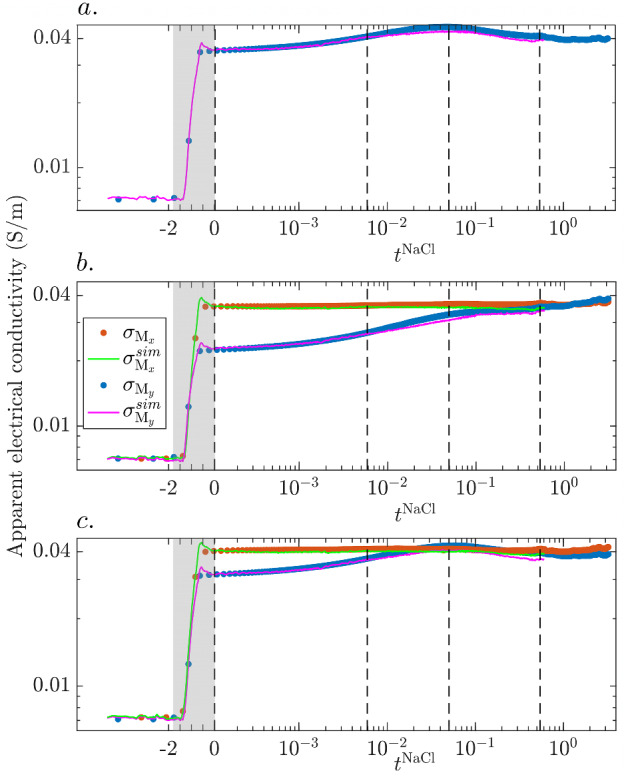
Measured (dots) and simulated (lines) time-series of apparent electrical conductivity under measurement modes M$_x$ (red and green) and M$_y$ (blue and magenta) for electrode pairs **a** $P_3$-$P_9$, **b** $P_4$-$P_5$ and **c** $P_4$-$P_9$. The four vertical black-dashed lines mark the times $t^{\text {NaCl}}_1 = 0$, $t^{\text {NaCl}}_2 = {5\times 10^{-3}}$, $t^{\text {NaCl}}_3 = {5\times 10^{-2}}$, and $t^{\text {NaCl}}_4 = {5\times 10^{-1}}$, respectively. Negative and positive times represent, respectively, times normalized by advective and diffusion transport time-scales (Sect. 3.3.2). The gray-colored rectangles highlight the tracer injection period extending over $60 \,\hbox {s}$

For the electrode pair $P_3$-$P_9$ (Fig. 6a), only the time-series corresponding to the measurement mode M$_y$ are plotted as the geometrical factor for measurement mode M$_x$ is above $5000 \,\hbox {m}$, which leads to highly unreliable apparent conductivities. Before the tracer injection period (highlighted by the gray-colored rectangle in Fig. 6a–c), the system is initialized and some electrical data are collected. For times $-1.8 \le t^{\text {NaCl}} \le -1.25$, the tracer injection has started, but Solution 2 is still saturating the inlet chamber (i.e, no tracer invasion into the porous medium) and the measured apparent conductivity (blue scatter in Fig. 6a) is approximately constant and equal to $\sim 0.07 \,{\hbox {S m}^{-1}}$. As the tracer invades the porous medium ($-1.25 \le t^{\text {NaCl}}\le 0$), the measured response shows a sharp increase by $\sim 5.5$ times the baseline apparent conductivity. After the injection is stopped, a smooth trend of increasing values is seen until $t^{\text {NaCl}}_3 = {5\times 10^{-2}}$ when the apparent electrical conductivity is $\sim 25 \%$ higher than the value at the end of the injection (at $t^{\text {NaCl}} = 0$). Subsequently, the apparent conductivities decrease slowly. The simulated apparent electrical conductivity time-series (magenta solid line) agrees well with the measured data although there is an overestimation of $\sim 7 \%$ for $-1.25 \le t^{\text {NaCl}} \le 0$. Also, the simulated data start to increasingly underestimate the data for $t^{\text {NaCl}} > t^{\text {NaCl}}_2$ reaching a maximal discrepancy of $\sim 5 \%$ at $t^{\text {NaCl}}_4$.

For electrode pair $P_4$-$P_5$ (Fig. 6b), the measured apparent conductivity time-series for injection configuration M$_y$ shows a different behavior compared to $P_3$-$P_9$. First, the tracer invasion leads only to an apparent conductivity increase in $\sim 3$ times the baseline value. Second, the positive growth rate spans the full duration of the experiment with a decreased rate from $t^{\text {NaCl}} > t^{\text {NaCl}}_3$. The simulated responses fit the data well, except for small over- and underestimations for times $-1.25 \le t^{\text {NaCl}} \le 0$ and $t^{\text {NaCl}} > t^{\text {NaCl}}_2$, respectively. Excepting the injection phase, the simulated response remains within $5.5 \%$ of the measured data. The measured apparent conductivity time-series for injection configuration M$_x$ shows a larger increase when the tracer invades the porous medium ($\sim 5$ times the baseline conductivity), and remains approximately constant during the rest of the experiment. The level of agreement of the simulated response with the measured data for M$_x$ shows a similar behaviour of over- and underestimation for the aforementioned time-periods. Apart from the overestimation during tracer injection ($\sim 7\%$), the simulated responses remain within $4 \%$ of the measured data.

The voltage time-series measured between $P_4$-$P_9$ for injection mode M$_y$ (Fig. 6c) behaves overall rather similarly to that of electrode pair $P_3$-$P_9$ (Fig. 6a). Around its maximum value, the apparent conductivity surpasses the one of M$_x$. For both injection modes, the simulated time-series show a very good agreement with the measurements, except for some over- and underestimation at early (i.e., $-1.25 \le t^{\text {NaCl}} \le 0$ during the tracer injection) and late (i.e., $t^{\text {NaCl}} > t^{\text {NaCl}}_2$) times, respectively. As for $P_4$-$P_5$, the apparent conductivity time-series for M$_x$ show more sensitivity to the tracer invasion than M$_y$ and remains rather flat afterwards.

## Discussion

### Proof of Concept and Technical Challenges

We have performed an electrically- and optically monitored milli-fluidic tracer test by injecting a solution of FS and NaCl in an artificial porous medium made of PDMS. Two key assumptions behind our approach are that: (i) the electrical conductivity time-series determined by the time-evolving NaCl concentration field can be modelled using the observed time-evolving FS concentration field by a suitable temporal and amplitude re-scaling; (ii) the PDMS material represents a porous medium that is quasi 2D, rigid and with negligible surface conductivity. In the following, we examine these two assumptions.

**Fig. 7:**
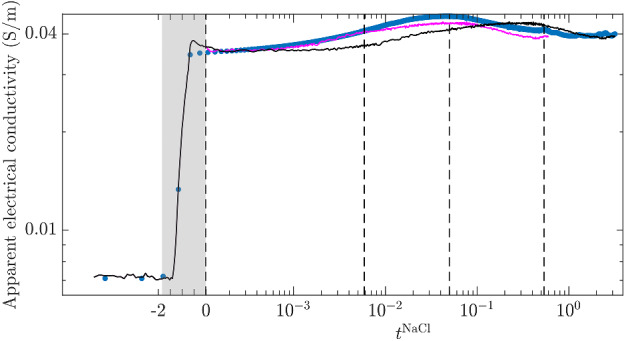
Apparent electrical conductivity time-series for the electrode pair $P_4$-$P_9$ and measurement mode M$_y$. Measured time-series (blue dots), simulated time-series with adequate time-scaling of FS concentration images to account for the different diffusion coefficients of FS and NaCl (magenta solid line) as described in Sect. 3.3.2 and without any such time-scaling (black solid line)

The simulated apparent conductivity time-series for electrode pair $P_3$-$P_9$ and injection type M$_y$ are plotted in Fig. 7 with and without temporal rescaling (see Sect. 3.3.2). This clearly highlights that simulated and observed data are best compared after accounting for the different diffusion coefficients of FS and NaCl salts.

The absence of advection after injection ($t^{\text {NaCl}} > 0$) implies that the diffusion coefficient *D* acts as a multiplicative constant in the governing transport equation (i.e., the diffusion equation). Thus, a ratio $D_{\text {NaCl}}/D_{\text {FS}} > 1$ can be compensated by a change of variables in the temporal coordinate such that the history of the unobserved NaCl concentration field is obtained from the time-compressed history of the observed FS concentration field. A previous related study Jougnot et al. (2018) implicitly assumed that the diffusion coefficients of both salts are equal, but this is only defensible when working at high Péclet numbers. One workaround at intermediate Péclet numbers is to design experiments for which the non-dimensional advection-diffusion equations for the FS and NaCl salts have the same Péclet number. To this aim, the salts would need to be advected by flow fields with mean flow velocities ${\bar{v}}$ differing by the ratio $D_\text {FS}/D_\text {NaCl}$. That is, one experiment with FS and another with NaCl needs to be carried out while ensuring the same ${\bar{v}}/D$ ratio.

On the one hand, the measured apparent electrical conductivity time-series appear rather insensitive to the observed apparent mass loss in the inferred concentration fields (Fig. 5). This insensitivity is particularly apparent for measurement mode M$_x$, for which the observed responses are close to flat after the injection (Fig. 6b, c). Given the approximately (*x*-parallel) layered distribution of tracer concentration, the bulk electrical conductivity is mainly sensitive to the arithmetic mean of the conductivities computed along *y*-oriented profiles and thus, mixing along *y* is expected to leave the time-series unmodified, provided that mass is conserved. On the other hand, the simulated data are impacted by the apparent mass loss in the inferred concentration fields, leading to an increasing underestimation of the observed data with time $t^{\text {NaCl}} > t^{\text {NaCl}}_2$. This growing inconsistency between observed and simulated data may indicate a small, but non-negligible, degree of FS photobleaching (e.g., Imamura and Koizumi 1955) leading to apparent FS mass loss. This seems to have occurred despite that we tried to decrease such effects using a flash lamp to diminish the exposure time of the tracer. The measured electrical data are mainly sensitive to the NaCl concentration and is, thus, basically insensitive to FS photobleaching. In future work, this effect could be corrected for by using a hydraulically isolated chamber that receives the same exposure over time.

Concerning the suitability of PDMS for this type of experiments, we highlight possible issues related to the tracer injection. At times $-1.25 \le t^{\text {NaCl}} \le 0$, we see that the simulated electrical responses overestimate the observed time-series of apparent electrical conductivity (Fig. 6a–c). We attribute this discrepancy to two possible PDMS-related sources of error. First, the slight flexibility of the acrylic plates used for sandwiching the PDMS (see Sect. 7.1) may have led to a slight inflation of the PDMS cell during injection, due to the applied pressure. In our calibration procedure and data processing, we assumed a rigid cell and thus a time-invariant cell thickness. For the considered range of FS concentrations, the light intensity is an exponentially increasing function of both the concentration and the cell thickness (e.g., Anna et al. 2014), implying that such an inflation could be misinterpreted as a slight increase in the concentration. Note that the measured conductivities should also be impacted by this effect, as they increase linearly with the height of the porous medium sample. However, the power-law dependence of concentration on light intensity, makes the fluorimetry data much more susceptible. Another possible error source during the injection period is optical effects appearing when the fluorescent tracer is invading the cell from left to right. During this period, the tracer acts as a moving light source that generates rapidly-changing patterns of light diffractions within the translucid PDMS material. Such patterns are manifested as secondary sources of light that might perturb the concentration estimates. In our calibration procedure, we are unable to account for such effects as a homogeneous distribution of the fluorescent tracer is assumed. Lastly, we confirm that the PDMS material has negligible surface conductivity, as evidenced by the linear log–log relationship in Fig. 2.

### Impact of Incomplete Mixing and Molecular Diffusion on Time-Series of Bulk Electrical Conductivity

#### Time-Series of Apparent Electrical Conductivity

The increased growth rate (or acceleration) of the measured apparent conductivity at $\sim t^{\text {NaCl}}_2$ (Fig. 6a–c) suggests that diffusion enhances the tracer connectivity by transporting mass from the middle high permeability channel towards the top and bottom low permeability channels of the porous medium (see modelled NaCl concentration profile in Fig. 4b). The maxima in all time-series at $\sim t^{\text {NaCl}}_3$ (Fig. 6a–c) suggest that at this moment the amount of NaCl mass brought by diffusion towards the sides is large enough to create a well-connected vertical path for the electrical current. Note that the responses reach their maxima $\sim 20$ times before the characteristic diffusive transport time-scale $\tau ^{\text {NaCl}}_\text {ch}$. This is simply a consequence of the impermeable boundaries located at $y=0$ and $y= 74 \,\hbox {mm}$, which increase the homogenization rate (see Fig. 4d). Since our initial tracer front is separated by a distance $w_{\text {ch}}$ from a wall, a representative time-scale for such confined situation is expected to be 10 to 50 times smaller than the one used $\tau ^{\text {NaCl}}_{\text {ch}}$ (e.g., Hamada et al. 2020).

**Fig. 8:**
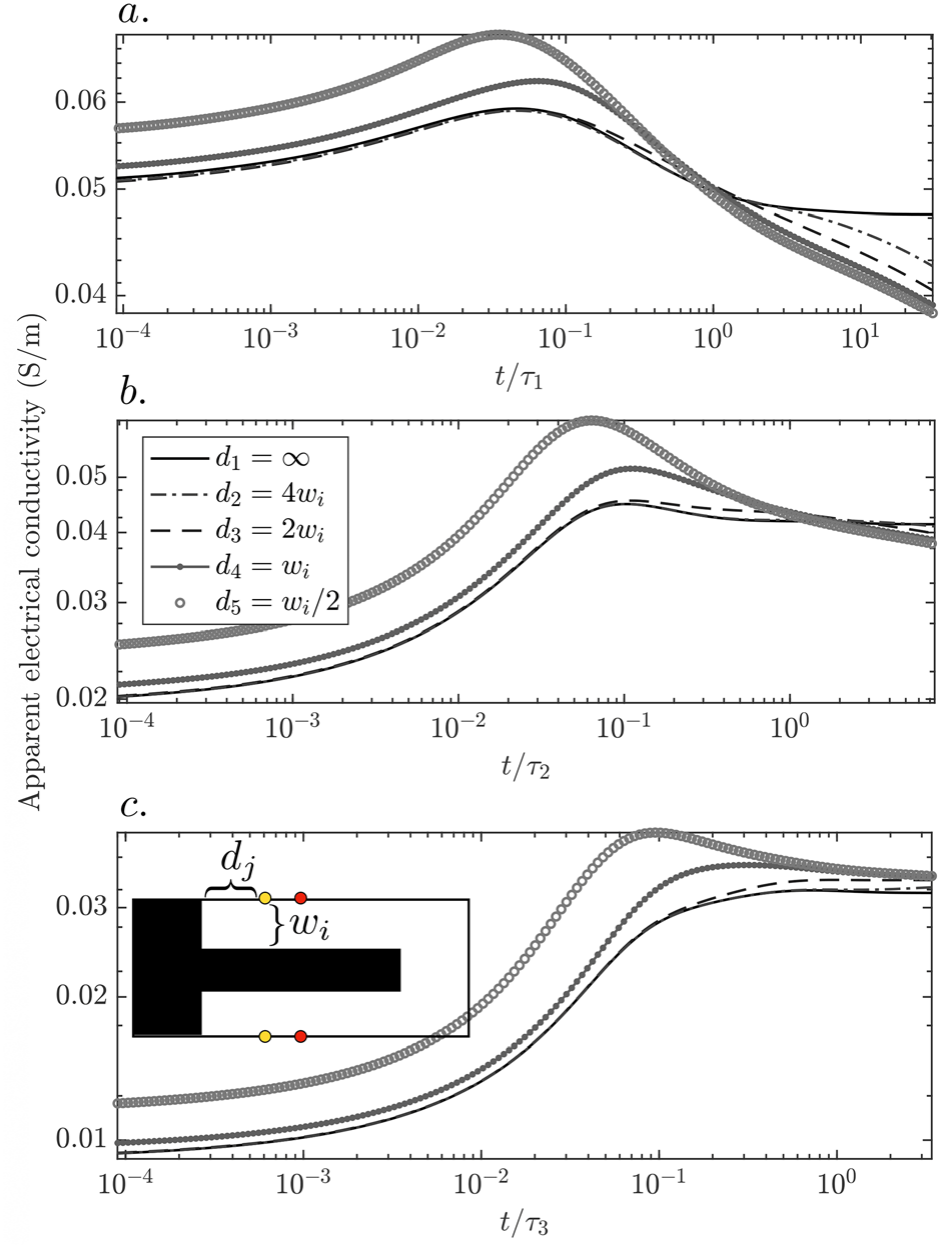
Simulated apparent electrical conductivity time-series for electrode pair $P_3$-$P_9$ under measurement mode M$_y$ for a cell that has top-bottom and middle layer widths of **a** $w_1 = 6 \,\hbox {mm}$ and $w^{\text {middle}}_1 = 62 \,\hbox {mm}$, **b** $w_2 = 12 \,\hbox {mm}$ and $w^{\text {middle}}_2 = 50 \,\hbox {mm}$ (as in the actual experiment) and **c** $w_3 = 18 \,\hbox {mm}$ and $w^{\text {middle}}_3 = 38 \,\hbox {mm}$. The time-series for each $w_i$ are plotted as a function of normalized times $t/\tau _i$, with $\tau _i = w_i^2/D_{\text {NaCl}}$ (defined analogously as $\tau ^{\text {NaCl}}_{\text {ch}}$ in sec. 3.3.1). A sketch illustrating the geometry of one of the initial NaCl concentration fields is shown in (**c**) along with the injection (red dots) and potential (yellow dots) electrodes $P_3$ and $P_9$

We now use numerical simulations to gain insights on the impacts of incomplete mixing and small-scale diffusion on the time-series of apparent electrical conductivity under injection mode M$_y$, using electrode pair $P_3$-$P_9$ as example. We examine different time-series resulting from the monitoring of solute plumes evolving from different initial widths and lengths until they reach complete mixing. Note that the considered initial conditions, inspired by the NaCl concentration field at $t^{\text {NaCl}}_1$ in the experiment (see the inset in Fig. 8c for a representation of the initial NaCl concentration field) could be viewed as a solute finger at the boundary of a large-scale solute plume. In such a setting, the bulk electrical conductivity at that location impacts ERT data during a field-scale experiment. Apart from the considered laboratory context, this suggests that our analysis may provide insight concerning the ability of ERT data to capture the mixing state and dynamics at the boundaries of solute plume bodies.

We simulate the time-evolving NaCl concentration field (Sect. 3.3) and the corresponding electrical conductivity time-series (Sect. 3.1). The modelling domain has unit porosity and the same dimensions as the experimental flow cell (see 2.1). Three different sets of initial conditions are considered, defined by the widths of the top and bottom layers, which are $w_1 = 6 \,\hbox {mm}$, $w_2 = 12 \,\hbox {mm}$ and $w_3 = 18 \,\hbox {mm}$. Since the modelling domain has the same height as the experimental flow cell, the associated middle layer widths $w^{\text {middle}}_i$ ($= 74 \,\hbox {mm} - 2 \times w_i$) are $w^{\text {middle}}_1 = 62 \,\hbox {mm}$, $w^{\text {middle}}_2 = 50 \,\hbox {mm}$ and $w^{\text {middle}}_3 = 38 \,\hbox {mm}$. For each set we place rectangular and identical chambers to the left and right sides of the domain, which are saturated with tracer (as the middle layer) and background (as the top-bottom layers) fluid, respectively (as in the experiment). For each layer width $w_i$, we vary the chambers’ sizes such that the left chamber has six different distances to the electrode pair $P_3$-$P_9$. These distances are $d_1 = \infty$ (1-D or layered NaCl concentration field), $d_2 = 4 \times w_i$, $d_3 = 2 \times w_i$, $d_4 = 1 \times w_i$ and $d_6 = 0.5 \times w_i$. We run the transport simulations for 8 days (twice the time of the actual experiment) in order to ensure an impact of the chambers for all the considered values of $d_j$. The tracer and background fluids are chosen to have electrical conductivities of $\sigma _{\text {S}_1} = 0.0218$ and $\sigma _{\text {S}_2} = 0.2728 \,{\hbox {S m}^{-1}}$, respectively (Sect. 2.7). Lastly, note that in the actual experiment, we had $w = w_2$ ($\equiv w_{\text {ch}}$, Sect. 3.3.1), $w^{\text {middle}} = w^{\text {middle}}_2$ and $d = 12 \times w_2$.

**Fig. 9:**
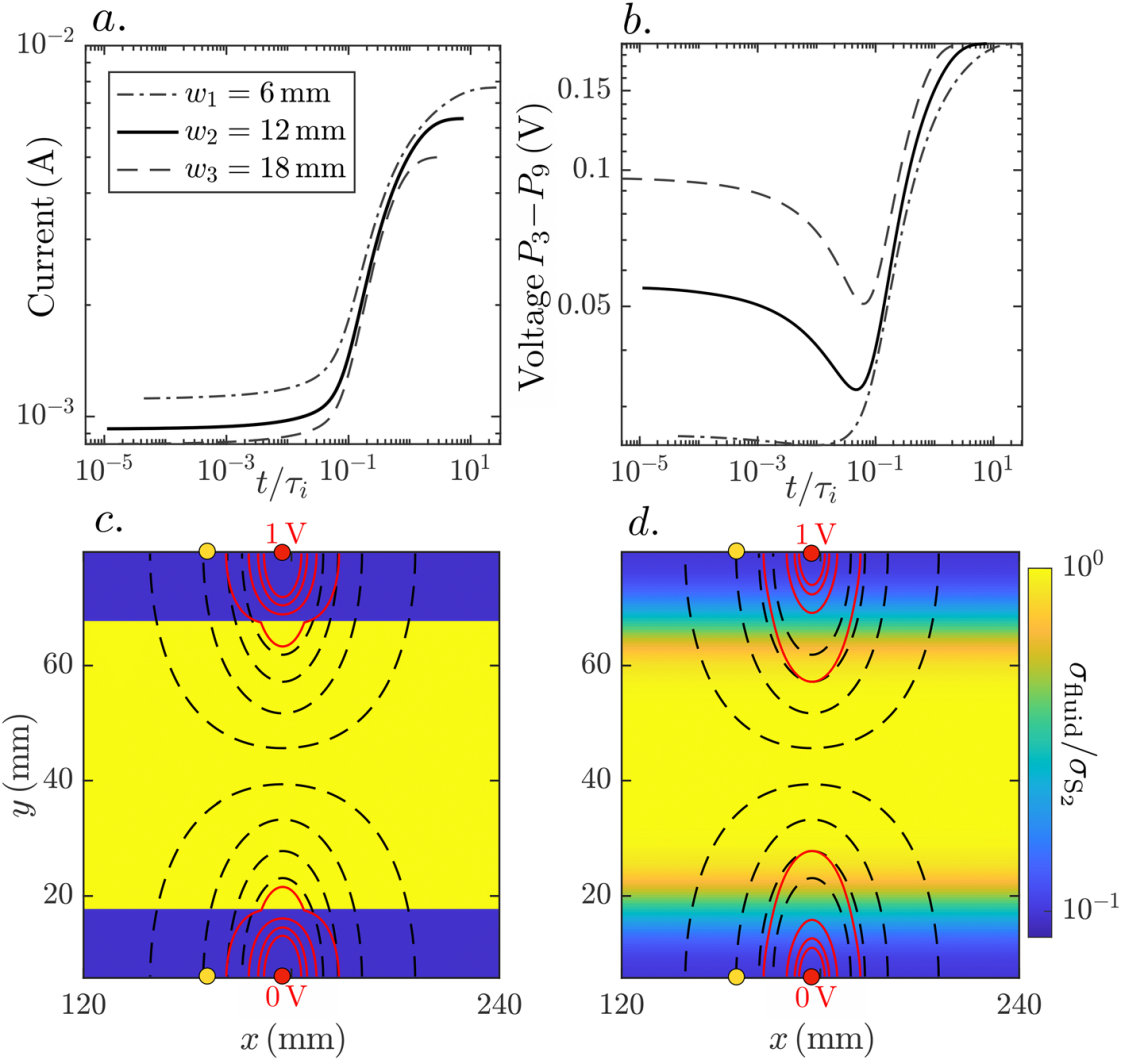
Simulated time-series of **a** total current flowing through the domain and **b** voltage measured between $P_3$-$P_9$, for chambers located at $d_1 = \infty$ (see the main text) and the three considered initial top and bottom widths of $w_1 = 6 \,\hbox {mm}$, $w_2 = 12 \,\hbox {mm}$ and $w_3 = 18 \,\hbox {mm}$. The response associated to $w_i$ ($i=1,2,3$) is plotted as a function of the corresponding normalized $t/\tau _i$ (see Fig. 8c–d) Simulated fluid conductivity (tracer concentration) field, normalized by $\sigma _{\text {S}_2}$, for $w_2 = 12 \,\hbox {mm}$ and $d_1 = \infty$ at (c) $t/\tau _{2} = 0$ and (d) $t/\tau _{2} = 0.1$. On top of each fluid conductivity field, eight equipotential lines of the electrical potential field for the levels $0.4 + 0.025 \times p \,\hbox {V}$ ($p=0,\ldots,3,5,\ldots ,8$) are shown for the corresponding conductivity field (red solid lines) and for the case of a homogeneous conductivity field (black dashed lines). The injection electrodes are indicated (red dots) as well as the potential electrodes $P_3$ and $P_9$ (yellow dots)

For $w_1$ (Fig. 8a), the apparent electrical conductivity time-series shows higher values at intermediate times (unmixed tracer) compared to late times (mixed tracer) for all $d_j$. This may appear counter-intuitive when considering $d_1 = \infty$ as diffusion along *y* increases the total current flowing through the sample (Fig. 9a) by connecting the top and bottom injection electrodes. This “overshoot” in apparent conductivity occurs by virtue of the perturbation of the electrical potential field, generated by the presence of the (horizontal) conductive tracer front, which modifies the voltage $P_3$-$P_9$. At an interface separating media of different conductivity, the tangential component of the electric field and the normal component of the current density field are continuous. These boundary conditions imply that the current density field lines, perpendicular to the equipotential lines, are refracted when the former cross the boundary between two bodies of different conductivity (e.g., Feynman et al. 2011). Such lines bend away from (towards to) the concentration gradient direction when they enter (exit) a more conductive body. In our setting, this is manifested in the electrical potential field as an overall compression of the equipotential lines (Fig. 9c). In particular, the compression of these lines along the *x*-direction leads to a decrease in the measured voltage (Fig. 9b), that translates into an increase in apparent electrical conductivity.

For $d_j \ge w_1$ ($j =1,\ldots ,4$), the time-series are maximized at $t/\tau _1 \sim 0.1$. As described above, the tracer arrival by diffusion leads to further compression of the equipotential lines along *x* (compare equipotential lines of Fig. 9c–d), which leads to a minimum of the voltage time-series (Fig. 9b), that in turn is manifested as a maximum in the conductivity time-series. The situation is different for $d_5$ ($< w_1$), for which the left chamber is closer to $P_3$-$P_9$ than the initial tracer front. Consequently, the time-series is maximized due to the (earlier) arrival of the tracer from the chamber.

For $w_2$, the starting values of the apparent conductivities are smaller than the final ones. Correspondingly, the voltage measurement from $P_3$-$P_9$ starts at a larger value when compared to the case for $w_1$. Additionally, note that for $t/\tau _2 \sim 3.7$, which corresponds to 4 days, that is, the duration of the actual experiment, the apparent electrical conductivity time-series for $d_2$ show only a negligible difference compared to the data for the 1-D case. Considering that for the PDMS cell, the chambers are located much farther apart than $d_2$, this indicates that for the experiment, the impact of diffusion from the chambers is negligible.

For $w_3$, the starting values of the apparent conductivities are again smaller than the final ones, although the difference is more marked than for $w_2$. Also, note the absence of a clear maximum occurring around $t / \tau _3$. For larger $w_i$, the tracer arrives more mixed to the electrodes at $t/\tau _i \sim 0.1$. Consequently, the associated perturbation of the electrical potential is less marked, hence, the less pronounced signature.

The simultaneous dependence of the time-series of apparent electrical conductivity on both the current and the voltage time-series (Fig. 9) makes such data difficult to interpret (e.g., Jung et al. 2009). However, it also enhances the information content concerning the temporal evolution of the spatial organization of the tracer. An example is the demonstrated sensitivity to the tracer arrival at the diffusion transport time-scale for a confined sample. Also, the ratio of the initial to terminal values of apparent conductivity is sensitive to the distance of the tracer front to the injection electrodes. In order to effectively exploit this information, it is necessary to develop a framework that quantitatively link solute transport-driven conductivity variations with perturbations of the electrical potential field. This will be the topic of future research.

#### Time-Series of the Equivalent Electrical Conductivity Tensor

**Fig. 10:**
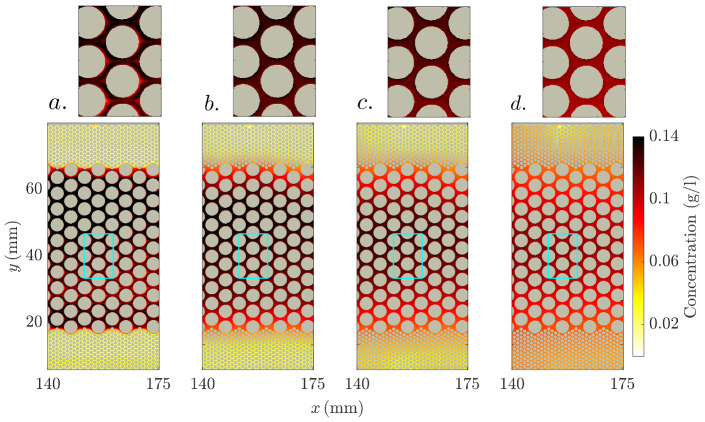
Four snapshots of the image-inferred NaCl concentration field contained in the sub-region defined by the horizontal coordinate range $x \in [140 , 175] \,\hbox {mm}$ at the times **a** $t^{\text {NaCl}}_1 = 0$, **b** $t^{\text {NaCl}}_2 = {5\times 10^{-3}}$, **c** $t^{\text {NaCl}}_3 = {5\times 10^{-2}}$ and **d** $t^{\text {NaCl}}_4 = {5\times 10^{-1}}$. The close views shown on top of the full concentration maps correspond to the areas enclosed by the cyan-colored rectangles in the corresponding full maps

We examine now the impact of the mixing of an initially layered concentration distribution on the time-series of the equivalent electrical conductivity tensor $\underline{{\underline{\sigma }}}$. We simulate the latter using as input fluid conductivity fields derived from the fluorimetry-inferred NaCl concentration fields (as for Sect. 4). However, we consider only a subregion of the images, from which four snapshots are shown in Fig. 10, in order to work with conductivity fields which are as layered as possible.

**Fig. 11:**
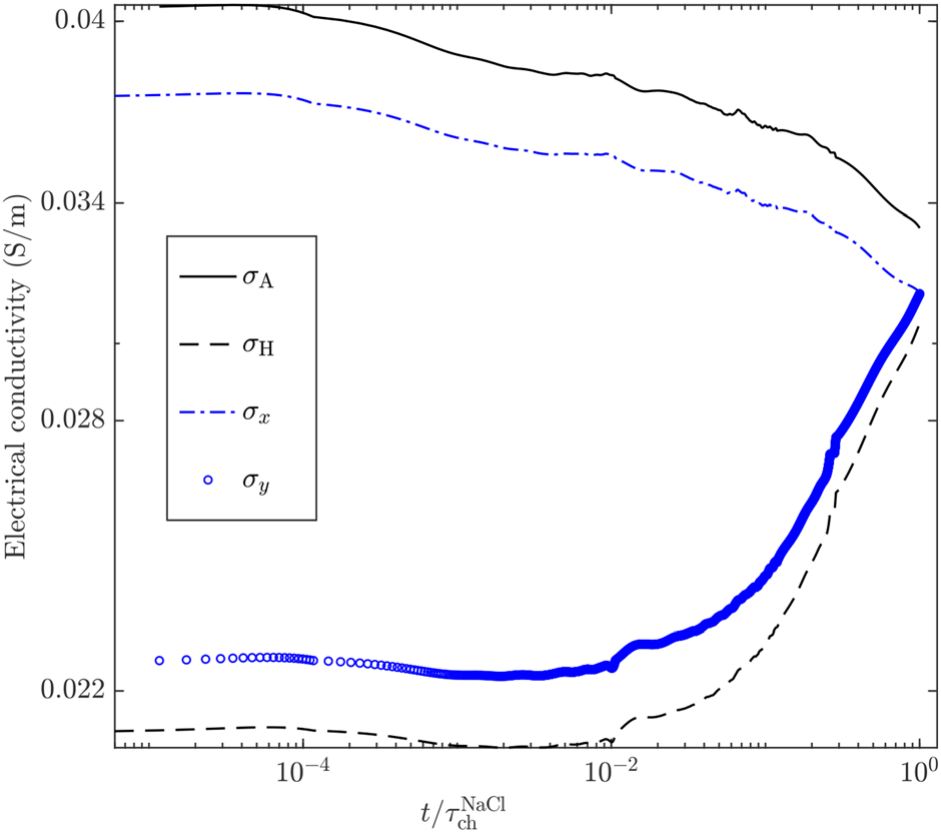
Simulated time-series of the *x* and *y* components of the equivalent electrical conductivity tensor, $\sigma _x$ and $\sigma _y$, and computed time-series of the arithmetic and harmonic means $\sigma _\text {A}$ and $\sigma _\text {H}$ over the considered sub-region (Fig. 10) of the time-evolving NaCl concentration field

We compute numerically the electrical potential as described in Sect. 3.1. However, we simulate line electrodes along the left and right (top and bottom) boundaries of the cell, respectively, for computing the components of $\underline{\underline{\sigma }}$. As before, for each excitation mode, a no-flux condition is imposed to the electrical potential along the other boundaries. Due to the *x*-oriented layering, the spatial coordinate system given by (*x*, *y*) is oriented along the principal directions of $\underline{\underline{\sigma }}$, implying that only its diagonal components, denoted by $\sigma _{x}$ and $\sigma _{y}$, are non-zero. For reference, we also compute the time-series of the Wiener bounds of the domain, that is, the harmonic $\sigma _\text {H}$ and arithmetic $\sigma _\text {A}$ means of the conductivity fields, divided by the (spatially constant) formation factor *F* (${:}{=}F_{\text {sim}}$, Sect. 3.2):

6 $$\begin{aligned} \sigma _\text {H} = \frac{1}{F}\frac{N}{\sum _{n=1}^{N} \frac{1}{\sigma _{\text {fluid}}^i}} , \end{aligned}$$

and

7 $$\begin{aligned} \sigma _\text {A} = \frac{1}{F}\frac{1}{N} \sum _{n=1}^{N} \sigma _{\text {fluid}}^i, \end{aligned}$$

where *N* and $\sigma _{\text {fluid}}^i$ denote, respectively, the number of pixels and the value of the fluid conductivity for pixel *i*. The computed time-series are shown in Fig. 11.

The *x*-oriented layered tracer distribution results in qualitative behaviours of $\sigma _{x}$ and $\sigma _{y}$ that are very close to those of $\sigma _\text {A}$ and $\sigma _\text {H}$, respectively. The discrepancies between $\sigma _{x}$ and $\sigma _\text {A}$, and between $\sigma _{y}$ and $\sigma _\text {H}$ indicate that the tracer distribution within the sub-sector is not perfectly layered (i.e., 1-D), as discussed previously. Note again the decay over time of $\sigma _\text {A}$ (and $\sigma _{x}$), indicative of apparent tracer mass loss within the considered sub-region (see Fig. 5 and Sect. 5.1). By the end of the experiment, $\sigma _{x}$ and $\sigma _{y}$ collapse at $\sim 0.03 \,{\hbox {S m}^{-1}}$ before the tracer has completely mixed (evidenced by the remaining separation between $\sigma _\text {A}$ and $\sigma _\text {H}$ at late times). At this point, the steep slopes of the conductivity time-series suggest that $\sigma _{y}$ would surpass $\sigma _{x}$ if longer times would have been considered. This is a consequence of the *x*-oriented gradient (see Fig. 5). Again, this is possible since the concentration distribution is not perfectly layered.

The time-evolution of the mean tracer concentration within the sample can be estimated from Archie’s law (Eq. 2), by inputting either $\sigma _{x}$ or $\sigma _{y}$ as the formation conductivity. When compared against $\sigma _\text {A}$ (i.e., the mean fluid electrical conductivity that is directly related to the mean tracer concentration), they yield solute mass recoveries of 92 and $60 \%$ at early times, respectively. Indeed, when using such an approach, apparent loss of mass is expected as soon as the tracer is not completely mixed within the sampled volume (Jougnot et al. 2018; Visentini et al. 2020) or measurements are made in the direction of stratification. This is a common problem arising during hydrogeophysical experiments, and is unrelated to the previously mentioned (optical) apparent tracer mass loss (see Sect. 5.1). These considerations may become relevant for anisotropic ERT experiments for which spatial distributions of both $\sigma _{x}$ and $\sigma _{y}$ are recovered (e.g., Herwanger et al. 2004).

The time-series for $\sigma _{y}$ shows an overall increase over time and no maximum at $t/\tau ^{\text {NaCl}}_{\text {ch}} \sim 0.1$ as described in Sect. 5.2.1. With line electrodes in a layered 2-D media, the potential lines are straight. Consequently, sensitivity to the temporal evolution of electrical potential perturbations is less evident, although it may still be captured by differentiating the time-series with respect to time (see Visentini et al. 2020).

Finally, we explore the relationship between the time-series of $\sigma _{y}$ and the temporal evolution of two common descriptors characterizing the degree of mixing: the concentration variance $\sigma _c^2$ and the scalar dissipation rate $\chi$ (e.g., Le Borgne et al. 2010). The latter is defined as

8 $$\begin{aligned} \chi = \int d\mathbf{x} {\nabla c}^T D \nabla c. \end{aligned}$$

**Fig. 12:**
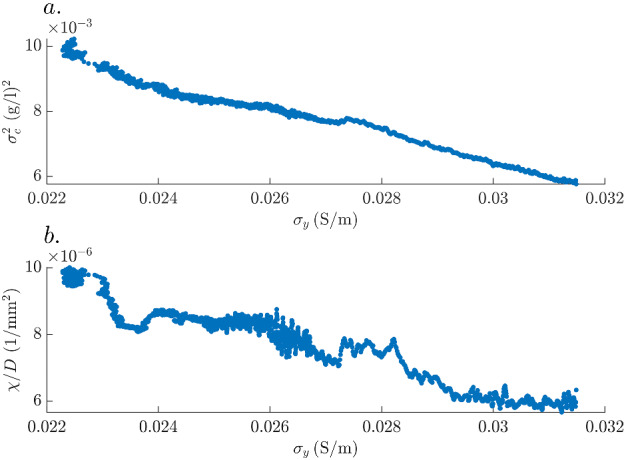
Scatter plots of the *y*-component of the equivalent electrical conductivity tensor, $\sigma _{y}$, plotted against **a** the concentration variance $\sigma _c^2 (t)$ and **b** the scalar dissipation rate divided by the diffusion coefficient, $\chi (t)/D$

In Fig. 12a–b, scatter plots of $\sigma _{y}$ versus $\sigma _c^2 (t)$ and $\chi (t)/D$ demonstrate strong negative correlations. We attribute the higher variability in Fig. 12b to noise in the image-recovered concentration gradient field rather than to variability in the relationship itself. Establishing quantitative links between statistical descriptors of the mixing state of the solute such as the concentration variance and the scalar dissipation rate with the time-evolution of the electrical conductivity are the topic of ongoing research.

## Conclusions

We have performed an optically- and electrically monitored milli-fluidic tracer test to study the electrical signatures of diffusion-limited mixing of an initially layered tracer distribution. We have confirmed that fluorimetry- and geoelectrically inferred time-series of apparent electrical conductivity can, in a diffusion-dominated environment, be related by a temporal re-scaling that accounts for the ratio of $\sim 3.8$ between the FS (optical) and NaCl (electrical) tracer diffusion coefficients. After this correction, we find that the observed and simulated apparent electrical conductivities are in strong agreement and demonstrate high sensitivity to the initial tracer invasion and subsequent diffusion. Particularly, the apparent electrical conductivity time-series measured perpendicularly to the concentration gradient are consistently maximized at times corresponding to the NaCl diffusion transport time-scale associated with the layer width in confined media. Numerical simulations confirm this and indicate high sensitivity of the electrical data to the layers’ degree of mixing and their distance to the injection electrodes. The time-evolving equivalent electrical conductivity in the direction of layering is strongly anti-correlated to two common solute mixing descriptors: the concentration variance and the scalar dissipation rate. In summary, our study provides a proof of concept for a novel experimental approach while pointing towards interesting avenues for establishing quantitative links between the mixing dynamics of the solute and time-series of electrical responses.

## Data Availability

The data collected during the experiment presented are stored in Zenodo (10.5281/zenodo.4066432) and are publicly available.

## Appendices

### Appendix A: Porous System Fabrication

The porous network is mechanically drilled into a stainless steel rectangular plate with $0.4 \,\hbox {mm}$ depth. A PDMS solution and curing agent (9:1 ratio) are then poured into the mold and left to cure at room temperature during $\sim 48$ h. Subsequently, the solid PDMS is detached from the mold and placed between two $2 \,\hbox {cm}$-thick transparent acrylic plates and the sandwich is tightened using mechanical clumps. Prior to assembling, the acrylic plate that is placed in contact with the positive reliefs of the cell is covered with a thin film of the same PDMS solution and both the reliefs and the film are radiated with plasma (e.g., Xiong et al. 2014) in order to ensure a high degree of cohesion of the cell.

### Appendix B: Geoelectrical Monitoring System

The geoelectrical monitoring system consists of two injection circuits and one measurement circuit, all operated using the Campbell Micrologger CR3000. The system is electrically connected to the flow cell using stainless steel cylindrical electrodes of diameter $1 \,\hbox {mm}$ that are pinched into the PDMS along the cell’s contour (Fig. 1) through holes in the top acrylic plate.

The injection circuits, connected to the cell using the sets of injection electrodes $A_x$-$B_x$ or $A_y$-$B_y$, are full resistive bridges comprising (i) a voltage source of amplitude $1 \,\hbox {V}$, connected in series with (ii) a bridge resistor of resistance $26700 \,\varOmega \pm 1\%$, (iii) the flow cell, which is the time-varying resistance of interest and (iv) another bridge resistor identical to the first one. These circuits are alternatively activated using a voltage switch relay, controlled by a logic port of the CR3000, to apply a voltage square wave of period $12 \,\hbox {s}$ formed by 4 cycles of $3 \,\hbox {s}$ each with the following sequence: $1 \,\hbox {V}$, $0 \,\hbox {V}$, $-1 \,\hbox {V}$ and $0 \,\hbox {V}$. This injection protocol alternating the polarity of the signal, standard for geoelectrical monitoring applications, is used to prevent cumulative electrode polarization and correct for possible electrode drift.

The measurement circuit consists of 13 high impedance differential voltage measurement channels that collect data for each injection mode. The voltage measurements are performed using the CR3000 “VoltDiff” instruction with (i) signal integration over $3 \,\hbox {ms}$, in order to remove $60 \,\hbox {Hz}$ noise, and (ii) polarity reversal, in order to remove any voltage offset coming from the datalogger circuitry. The CR3000 needs $\sim 0.65 \,\hbox {s}$ to perform the 13 measurements. The recorded voltages are obtained by averaging the measured voltage during the positive and negative excitation cycles mentioned above. The individual measurements for each cycle are taken $2.3 \,\hbox {s}$ after initiating each current injection to allow the recorded signal to stabilize.

### Appendix C: Grain Mask Compilation

We binarize a light intensity image that displays a high intensity contrast between the pore space and the solid phase; it is obtained by saturating the medium with Solution 2. We use a spatially variable binarization threshold value obtained from Otsu’s thresholding algorithm Otsu (1979) within subwindows of $20 \times 20$ pixels. A local thresholding is preferred due to the spatially varying light intensities. The binarization distorts the shape of the originally circular grains somewhat. To avoid this, the binary image is used to locate the centroids of all the (distorted) grains. They are then replaced by circular disks of required size. In this way, known internal distances within the lattice are preserved.
